# Comparison of two Crimean-Congo haemorrhagic fever virus serological tests in Tanzanian febrile patients using a Bayesian latent class approach

**DOI:** 10.3389/fmicb.2026.1894712

**Published:** 2026-10-16

**Authors:** Elichilia R. Shao, Rebecca F. Bodenham, Jaffu Chilongola, Sarah Cleaveland, Venance P. Maro, Buliga Mujaga, Eliaichi A. Mlay, Laura J. Shirima, Jeremia J. Pyuza, Judith Njau, Felix Lankester, Ray Kayaga, Matthew P. Rubach, John A. Crump, Chee Wah Tan, Lin-Fa Wang, Melinda K. Rostal, Blandina T Mmbaga

**Affiliations:** 1 Internal Medicine Department, Kilimanjaro Christian Medical Center, Moshi, Tanzania; 2 Kilimanjaro Clinical Research Institute, Moshi, Tanzania; 3 Internal Medicine Department, School of Medicine, KCMC University, Moshi, Tanzania; 4 Department of Medical Biochemistry and Molecular Biology, School of Medicine KCMC University, Moshi, Tanzania; 5 School of Biodiversity, One Health and Veterinary Medicine, University of Glasgow, Glasgow, United Kingdom; 6 Department of Prosthetics and Orthotics, School of Rehabilitation Medicine, KCMC University, Moshi, Tanzania; 7 Department of Epidemiology and Biostatistics, School of Public Health, KCMC University, Moshi, Tanzania; 8 Pathology Department, School of Medicine, KCMC University, Moshi, Tanzania; 9 Global Health Tanzania, Arusha, Tanzania; 10 Paul G. Allen School for Global Health, Washington State University, Pullman, WA, United States; 11 Department of Life Sciences and Bio-Engineering, Nelson Mandela African Institution for Science and Technology, Arusha, Tanzania; 12 Tanzania Veterinary Laboratory Agency, Arusha, Tanzania; 13 Duke Global Health Institute, Duke University, Durham, NC, United States; 14 Division of Infectious Diseases and International Health, Duke University Medical Center, Durham, NC, United States; 15 Centre for International Health, University of Otago, Dunedin, New Zealand; 16 Programme in Emerging Infectious Diseases, Duke-National University of Singapore Medical School, Singapore, Singapore; 17 One Health Research Consulting, NJ, United States; 18 Department of Production Animal Studies, Faculty of Veterinary Science, University of Pretoria, Onderstepoort, South Africa; 19 Pediatric and Child Health Department, School of Medicine, KCMC University, Moshi, Tanzania

**Keywords:** Bayesian latent class model, Crimean-Congo haemorrhagic fever virus, enzyme-linked immunosorbent assay, fever, serology, Tanzania

## Abstract

**Background:**

Crimean-Congo haemorrhagic fever virus (CCHFV) is a tick-borne zoonotic disease that can cause severe disease in humans, has a high fatality ratio, and is present in many African countries. Accurate diagnosis of CCHF remains difficult. Serological and molecular tests are frequently used to detect exposure or infection, but their accuracy varies with viral strain, infection stage, and population factors. Notably, there is no universally recognised gold standard test for CCHFV serological diagnosis. The reference standard for antibody detection requires neutralisation assays, which must be performed in biosafety level 4 (BSL-4) laboratories. These facilities are scarce and largely unavailable in sub-Saharan Africa, including Tanzania, where CCHFV is endemic. This study aimed to evaluate the diagnostic performance of the IDvet ELISA and Luminex serological assays for detecting prior CCHFV exposure in humans using Bayesian latent class modelling in the absence of a reference standard.

**Methodology:**

This was a secondary analysis of archived serum samples from a prospective febrile illness surveillance cohort conducted between 2012–2014 in Moshi, Tanzania, at Kilimanjaro Christian Medical Center and Mawenzi Regional Referral Hospital. Bayesian latent class analysis was used to evaluate the performance of IDvet and Luminex assays in detecting CCHFV exposure in the samples collected.

**Results:**

Of the 870 febrile admissions enrolled across both sites, serum samples were available for 427 participants (49%). The median posterior estimate for IDvet sensitivity was 0.893 (95% Bayesian credibility interval (BCI) = 0.555–0.996) and specificity was 0.972 (95% BCI = 0.936–0.996). For the Luminex assay, the median posterior estimate for sensitivity was 0.884 (95% BCI = 0.541, 0.995) and specificity was 0.987 (95% BCI = 0.947, 1.00). The model generated seroprevalence estimates for two subpopulations, livestock owners and non-livestock owners. The livestock owner seroprevalence estimate was 0.027 (95% BCI = 0.002, 0.069), and for non-livestock owners, 0.017 (95% BCI = 0.001, 0.044).

**Conclusion:**

In the absence of a reference standard and access to BSL-4 neutralisation testing, both the IDvet ELISA and the Luminex assay demonstrated high diagnostic performance with comparable sensitivity in detecting prior exposure to CCHFV among febrile patients in northern Tanzania. Furthermore, the IDvet may offer operational advantages because of its simplicity and suitability for decentralised laboratories.

## Introduction

Crimean-Congo haemorrhagic fever virus (CCHFV) is a zoonotic virus prevalent across Europe, Asia, and Africa, that can pose a public health threat in endemic regions (Hoogstraal, 1979; Thompson et al., 2024; Zhioua et al., 2024). The virus can be transmitted via infected ticks as well as through contact with blood or tissues of an infected person or animal (Whitehouse, 2004; Akinci et al., 2013). *Hyalomma* species ticks are considered a reservoir for the virus (Ergönül, 2006; WHO, 2025). CCHFV can cause severe haemorrhagic fever in humans, with the fatality ratio ranging from 5 to 40% during outbreaks (Payne, 2017; Nasirian, 2020). Given its virulence, as well as the lack of effective antiviral treatments or vaccine, CCHFV is considered a high-priority pathogen in the WHO R&D Blueprint (Mehand et al., 2018; WHO, 2018). Despite the high fatality ratios reported during CCHF outbreaks, disease manifestations are highly variable, ranging from asymptomatic and subclinical infections to severe clinical illness(Aradaib et al., 2010; Adesola et al., 2023; Zalwango et al., 2024; Sene et al., 2025). In low-resource settings, clinical diagnosis is particularly challenging because early symptoms such as fever, headache, myalgia, arthralgia, nausea, and vomiting are nonspecific and overlap with many other febrile illnesses, including malaria, dengue, typhoid fever, leptospirosis, and Rift Valley fever(Crump et al., 2013; Chipwaza et al., 2014; Maze et al., 2018). This overlap substantially increases the risk of misdiagnosis and delayed detection (Seth et al., 2015; Maze et al., 2018; Rugarabamu et al., 2022). Incorporating systematic CCHFV testing into febrile illness etiology studies could substantially improve estimates of disease burden, transmission patterns, and early outbreak detection in endemic regions (Tigoi et al., 2015; Carugati et al., 2019; Blair et al., 2022). In line with findings in Uganda(Atim et al., 2023), studies in Tanzania have demonstrated human exposure and serological evidence of ongoing CCHFV circulation among livestock populations (Clare et al., 2022; Hughes et al., 2024), which suggest active transmission and circulation of the virus in Tanzania (Rugarabamu et al., 2022; Shao et al., 2025). However, to date no human cases of CCHF has been confirmed in Tanzania.

Despite being a severe and widely distributed tick-borne disease, public health action against CCHF remains hampered by major diagnostic gaps, including a lack of widely validated, standardised tests for human and animal surveillance (Mazzola and Kelly-Cirino, 2019; Sorvillo et al., 2020; Muzammil et al., 2024). Several commercial molecular and serological diagnostic tests are available for detection of CCHFV exposure and infection (WHO, 2024). These include nucleic acid amplification tests (NAATs), such as reverse transcriptase polymerase chain reaction (RT-PCR), or loop-mediated isothermal amplification (LAMP), as well as viral culture, and antibody detection tests (e.g., ELISA, IFA, and plaque-reduction neutralisation assays), including point of care lateral flow assays (WHO, 2023; Emmerich et al., 2021; Hawman and Feldmann, 2023). However, challenges remain with NAAT approaches due to the substantial genetic variation among viral lineages (Muzammil et al., 2024). Antibody-based serological assays are generally less impacted by genetic variation (Vanhomwegen et al., 2012), but also have limitations such as delayed antibody response, cross-reactivity, and inability to distinguish recent from past infection. Neutralisation assays are considered the reference standard for many arboviruses, but they require high-containment laboratory facilities, which are rarely available in low-resource settings, and are of limited utility in the diagnosis of CCHF as the virus elicits low levels of neutralising antibody (Mazzola and Kelly-Cirino, 2019). Despite the extensive range of commercial and in-house serological tests that are currently available for the detection of both IgM and IgG antibodies to CCHFV, challenges remain in relation to validation and standardisation (Messina et al., 2015; Mazzola and Kelly-Cirino, 2019; Shao et al., 2025).

The Hui-Walter Bayesian analysis model is a statistical framework for assessing the sensitivity and specificity of diagnostic tests in the absence of a gold standard. This model is commonly used in situations where the actual disease status of subjects is uncertain, such as in serological testing (Joseph et al., 1995; Chow and Hoijtink, 2017; Cheung et al., 2021). This study aimed to evaluate the diagnostic performance of the IDvet ELISA and Luminex serological assays for detecting prior CCHFV exposure in humans using a two-population Bayesian latent class model (BLCM) in the absence of a gold standard.

## Materials and methods

### Research clearance and ethics

The study was conducted in accordance with the ethical standards of the Declaration of Helsinki. The research protocol received approval from the Kilimanjaro Christian Medical University College Research Ethics Committee (#295), the Tanzania National Institute for Medical Research National Ethics Coordinating Committee (NIMR/HQ/R.8a/Vol. IX/1000), Duke University Medical Center Institutional Review Board (IRB#Pro00016134), the University of Otago Human Ethics Committee (Health) (H15/055), and the Duke-National University of Singapore Institutional Review Board. All eligible patients, or guardians for those aged <18 years, provided written informed consent to take part in the study.

### Study area

Data and sample collection for this analysis were conducted through a prospective febrile illness surveillance study aimed at identifying risk factors for bacterial zoonotic infections. The sample collection and analysis took place from 2012 to 2014 in Moshi, Tanzania, at Kilimanjaro Christian Medical Centre (KCMC) and Mawenzi Regional Referral Hospital (MRRH) (Crump et al., 2013; Maze et al., 2018a). At the time, KCMC was a 450-bed zonal referral and teaching hospital serving more than 15 million people in northern Tanzania. MRRH, five kilometres from KCMC, was a 300-bed hospital and the referral hospital for the Kilimanjaro Region. Moshi, the administrative capital of the Kilimanjaro Region, is situated at an elevation of 890 meters above sea level. The climate is tropical, characterised by a short rainy season from October through December and a longer rainy season from March to May or early June. Livestock production practices in Kilimanjaro region can broadly be classified as pastoral, agro-pastoral, and smallholder systems (Maze et al., 2018; Shao et al., 2025).

### Study design

The data originated from a previous prospective cohort study enrolling febrile patients between 2012–2014, and has been described previously (Maze et al., 2018a). Briefly, screening and enrolment of study participants occurred at the adult medical ward at KCMC, the adult and paediatric medical wards at MRRH, and the outpatient department at MRRH. Outpatients were eligible for enrolment if they had a tympanic temperature of ≥38 °C. Inpatients from the paediatric and medical wards were screened within 24 h of admission and were eligible if they had a tympanic temperature of ≥38 °C, or reported a fever within the past 72 h. Participants were followed up for resampling up to 6 weeks later. For the current study, the analysis was performed using the follow up samples only.

### Data and specimen collection

After obtaining informed consent, trained study personnel administered a standardised questionnaire with questions on demographics and exposures, including any activities involving direct or indirect contact with livestock. Venous blood was collected from participants into serum separator tubes, left to clot for 30 to 60 min, and then centrifuged at 1,000 g for 10 min to separate serum. Serum aliquots were stored at −70 °C ready for analysis. We excluded participants with insufficient serum sample volume or missing demographic data.

### Subpopulation definitions

The seroprevalence of CCHFV among livestock keepers and non-livestock keepers is expected to differ (McCabe et al., 2010; Maze et al., 2018b; Carugati et al., 2019; Bodenham et al., 2020). For the purpose of this study, the febrile patient population were classified as either livestock owners or non-livestock owners. Livestock owners were defined as participants stating that their household owned one or more livestock species (cattle, sheep, goats, pigs, poultry, camels, or horses). Non-livestock owners were individuals stating that their household did not own or raise any livestock.

### IDvet assay

Detection of IgG antibodies to CCHFV nucleocapsid protein (NP) was performed using the commercial ID screen® CCHF double-antigen multi-species enzyme-linked immunosorbent assay (ELISA) kit (IDVet innovative diagnostics, France) according to the manufacturer’s protocol. In brief, the test sera were diluted and incubated at 25 °C for 45 min. The conjugate and substrate steps were performed at 25 °C for 30 and 15 min, respectively, after which the reaction was halted. Absorbance was measured at 450 nm using an automated ELISA reader (BioTek ELx800, United States) with Gen5 version 2.06 software. The sample positivity percentage (S/P%) for each specimen was calculated by dividing the optical density (OD) of the sample (ODS) by the OD of the positive control (ODPC), expressed as a percentage. Serum samples were deemed positive if their S/P% exceeded 30% (Sas et al., 2018). We used Thermo Scientific™ Multiskan™ FC 96-well plate microplate photometers, serial number 357–913543.

### Luminex assay

Serologic evidence of exposure to CCHFV was likewise assessed using a laboratory-developed microsphere immunoassay (Luminex, Austin, TX, USA) for detection of CCHFV-NP, as described perviously (Shao et al., 2025). Test sera were screened using this qualitative, multiplex, magnetic bead–based assay in which virus-specific microsphere sets enabled simultaneous detection of NP-targeted IgG in a single well.

### Model assumptions

There are three key assumptions of the Hui-Walter model. The first assumption is that the sensitivity and specificity of each test under investigation are considered constant across the subpopulations being studied. In other words, the diagnostic performance of the tests does not change based on population, disease status, or other factors. We considered this assumption was not violated because the livestock keepers and non-livestock keepers tested were from the same geographic area, with broadly similar demographic and environmental conditions (Vacek, 1985; Toft et al., 2005).

The second Hui-Walter model assumption is that the diagnostic tests have the same sensitivity and specificity across subpopulations, despite differences in seroprevalence among subpopulations (Clare et al., 2022; Hughes et al., 2024). This assumption was not considered to be violated because samples were collected and processed, and diagnostic assays implemented, using standardised protocols. Additionally, laboratory personnel were blinded to the subpopulation status of participants. The potential for differences in test performance due to variation in geographic origin of study participants was also reduced, as all participants were from the catchment area of KCMC or MRRH in Moshi, Tanzania.

The third assumption is of conditional independence, where tests are presumed independent given the true disease status. For exposed individuals, testing positive on one test is independent of the results on the other; the same applies to non-exposed individuals testing negative. The Luminex assay is a multiplex technology that measures multiple antibodies related to hemorrhagic viruses in a single sample. It utilises fluorescence and flow cytometry principles to deliver quantitative and qualitative data (Hoste et al., 2023). The IDvet uses ELISA technology, specifically a double-antigen sandwich ELISA, to detect antibodies against CCHFV NP in multiple animal species and humans. Although these tests differ in their methodology of antibody detection, both tests measure the same underlying biological process, host antibodies to CCHFV antigens and their results are likely to be correlated, even after accounting for true infection status. Therefore, the assumption of conditional independence could not be met and consequently, the model was extended to incorporate conditional dependence using a covariance parameterisation as described elsewhere (Vacek, 1985; Plowright et al., 2021).

The probabilities (Pr) of observing each test result combination are defined as follows:

$\begin{array}{l} \mathrm{Pr}(T_{A}+T_{B}+)={{\text{prev}}_{i}}^{*}\;({{\mathrm{Se}}_{A}}^{*}{\mathrm{Se}}_{B}+\text{covEP})\\ +{\left(1-{\text{prev}}_{i}\right)}^{*}\;({\left(1-{\mathrm{Sp}}_{A}\right)}^{*}(1-{\mathrm{Sp}}_{B})+\text{covEN}). \end{array}$

$\begin{array}{l} \mathrm{Pr}(T_{A}+T_{B}-)={{\text{prev}}_{i}}^{*}\;({{\mathrm{Se}}_{A}}^{*}(1-{\mathrm{Se}}_{B})—\text{covEP})\\ +{\left(1-{\text{prev}}_{i}\right)}^{*}\;((1-{\mathrm{Sp}}_{A})*{\mathrm{Sp}}_{B}—\text{covEN}). \end{array}$

$\begin{array}{l} \mathrm{Pr}(T_{A}-T_{B}+)={{\text{prev}}_{i}}^{*}\;(\begin{array}{l} \left(1-{\mathrm{Se}}_{A}\right)\\ {}*{\mathrm{Se}}_{B}—\text{covEP} \end{array})\\ +{\left(1-{\text{prev}}_{i}\right)}^{*}\;({{\mathrm{Sp}}_{A}}^{*}(1-{\mathrm{Sp}}_{B})—\text{covEN}). \end{array}$

$\begin{array}{l} \mathrm{Pr}(T_{A}-T_{B}-)={{\text{prev}}_{i}}^{*}\;(\begin{array}{l} {\left(1-{\mathrm{Se}}_{A}\right)}^{*}(1-{\mathrm{Se}}_{B})\\ +\text{covEP} \end{array})\\ +{\left(1-{\text{prev}}_{i}\right)}^{*}\;({{\mathrm{Sp}}_{A}}^{*}{\mathrm{Sp}}_{B}+\text{covEN}). \end{array}$

where Se = sensitivity, Sp = specificity, A = IDvet, B = Luminex, covEP = represents the covariance between exposed participants, while covnEN = represents covariance among non-exposed participants, prev_i_ = denotes infection prevalence in the *i*th subpopulation.

### Prior distributions

A literature review was conducted to synthesise the current knowledge on diagnostic test performance of the IDvet and Luminex assays in detecting exposure to CCHFV in humans. Sensitivity and specificity values were extracted to inform prior distributions. The bias magnitude was assessed through a systematic review of each study’s quality before setting weights for the priors of sensitivity and specificity. Studies with lower bias risk had more influence, as their estimates were more reliable and reflective of true diagnostic performance.

The search included the following terms; “Crimean-Congo Hemorrhagic Fever Virus,” “CCHFV serological diagnosis,” “ID Screen® assay,” “Luminex immunoassay,” “CCHFV IgG/IgM detection,” and “seroprevalence studies.” Boolean operators (AND, OR) were used, and filters limited results to peer-reviewed articles published from January 2018 to December 2025 in English, as IDvet was first introduced in 2018. Special emphasis was placed on studies conducted in East Africa (e.g., Kenya, Uganda, Tanzania, Ethiopia, Sudan), and data from other endemic regions worldwide were also included due to the scarcity of studies in East Africa. Articles were included if they reported on the diagnostic performance (e.g., sensitivity, specificity) of these assays or provided seroprevalence data in human populations. References from key articles were screened for additional studies. Details of each study selected to provide sensitivity and specificity estimates for the IDvet and Luminex assays in detecting CCHFV exposure in humans are shown in Tables 1, 2.

**Table 1:** Mean sensitivity and specificity estimates and 95% confidence intervals for CCHFV diagnosis using luminex and IDvet assays in individuals from 2015 to 2025.

References	Diagnostic assay	Location	Sample size	Sensitivity (95% CI)	Specificity (95% CI)
Sas et al., 2018	IDvet	France Germany	257	0.989 (0.968–0.998)**	1 (0.998–1)
Wu et al., 2014	Luminex	China	182	NA	1 (0.980–1)

**Sensitivity was calculated using serum samples from multiple species, not including human samples. CI is confidence interval.

**Table 2:** Reported seroprevalence of CCHFV in humans in Eastern Africa from 1967 to 2025.

Reference	Diagnostic test	Location	Sample size	Seroprevalence (%)
Rugarabamu et al., 2021	IgG-IgM ELISA (My BioSource, Inc., San Diego, CA, USA)	Tanzania	500	2.0
Lwande et al., 2012	IgG ELISA (VectoCrimean-CHF-IgG and IgM ELISA test kits; Vector-Best, Novosibirsk, Russia).	Kenya	517	19.0
Nyataya et al., 2020	IgG ELISA (VectoCrimean-CHF-IgG kit; Vector-Best, Novosibirsk, Russia)	Kenya	1,033	1.9
Mihalakakos et al., 2025	IgG ELISA (IDvet, ID Screen® CCHF Double Antigen ELISA; Innovative Diagnostics, Grabels, France)	Uganda	1,200	7.8
Lule et al., 2022	IgG ELISA (VectoCrimean-CHF-IgG and IgM ELISA test kits; Vector-Best, Novosibirsk, Russia)	Uganda	478	10.3
Hughes et al., 2024	IgG ELISA (IDvet, ID Screen® CCHF Double Antigen ELISA; Innovative Diagnostics, Grabels, France)	Tanzania	351	15.1
Shao et al., 2025	IgG ELISA (IDvet, ID Screen® CCHF Double Antigen ELISA; Innovative Diagnostics, Grabels, France)	Tanzania	735	1.8

Sensitivity and specificity priors assumed a beta distribution with shape parameters (α, β). For each sensitivity or specificity prior, because literature estimates were sparse we generated priors that reflected the direction of the available literature while allowing sufficient uncertainty for the observed data to meaningly influence posterior estimates. For the Luminex sensitivity, no literature was identified. Therefore we initially considered a non-informative prior (1,1), which we did not adopt as it resulted in a posterior estimate with extremely wide BCIs, indicating that the data were insufficiently informative. Since the BLC models for two diagnostic tests’ performance without a gold standard infer true disease status jointly with prevalence and test performance parameters, the estimate of sensitivity and its precision depend on the number of subjects providing strong evidence of true positivity (double-positive results), rather than the overall sample size. With only 7 double positives, this limited informative sample resulted in greater posterior variation and hence wider credibility intervals for sensitivity. In the final model we specified the Luminex sensitivity prior the same as the IDvet prior, given the comparable serological detection principles of the two assays.

We identified in East Africa, CCHFV seroprevalence estimates ranging from 1.8 to 19% (Table 2). Guided by the literature, we used an informative beta distribution with a prior mean of 9.1%, which approximated the sample-size-weighted pooled seroprevalence estimate of 7.0% from these studies. This distribution assigns greater probability to lower prevalence values while retaining reasonable support for the higher values reported in the literature.

For each Beta (α, β) prior, the mean was calculated as α/(α + β), representing the expected value of the parameter before observing the study data, while the effective prior sample size was calculated as α + β, representing the number of “pseudo-observations” of information the prior contributes relative to the observed data. Sensitivity, specificity, and seroprevalence priors are listed in Table 3.

**Table 3:** Prior beta distribution shape parameters (*α*, *β*) for sensitivity and specificity of the IDvet and luminex assays.

Test and parameter	Beta distribution (*α*, *β*)
IDvet sensitivity	(6.5, 1)
IDvet specificity	(2, 1)
Luminex sensitivity	(6.5, 1)
Luminex specificity	(2, 1)
Disease seroprevalence	(1, 10)

### Model implementation and analysis of posteriors

For model implementation, we assigned prior distributions to Se_A_, Se_B_, Sp_A_, Sp_B_, and seroprevalence based on existing literature. The likelihood function was defined as the probability of the observed data given these parameters. For posterior inference, Bayesian methods were used to estimate the distributions of the parameters by combining the priors with the observed data. This process was carried out using Markov Chain Monte Carlo (MCMC) techniques (Srivastava and Kumar, 2011).

A Bayesian model in Just Another Gibbs Sampler (JAGS) was implemented using the rjags package in R (Qi et al., 2022). We selected the number of iterations and posterior samples to optimise computational efficiency without compromising convergence. The final model settings included three chains, a burn-in phase of 10,000 iterations, and 6,000 sampling iterations thinned by 10, resulting in 1,800 posterior samples. Model convergence was assessed with the Gelman-Rubin diagnostic (R-hat), where an R-hat value of ≤ 1.1 for all parameters indicates successful convergence and an effective sample size ≥400 (the minimum threshold), and most exceeded 1,500. The Heidelberger–Welch (H-W) stationarity test passed for all parameters; a few converged after discarding a few early iterations, but they ultimately passed the test, providing evidence of convergence (Vehtari et al., 2022). Additionally, trace plots were used for visual inspection of chain mixing. We also assessed the effective sample size (ESS), aiming for an ESS > 200 for each parameter, to indicate reliable posterior estimates. The positive predictive value (PPV) and negative predictive value (NPV) were also calculated for each diagnostic assay. After confirming convergence, we summarised the posterior distributions to obtain point estimates and measures of uncertainty. Posterior median and 95% BCI were reported for the sensitivity and specificity of each test, as well as for the true seroprevalence within each subpopulation. All analyses were conducted using R version 4.4.1.

## Results

Of 870 febrile individuals, 427 (49.1%) had serum available for both IDvet and Luminex assay testing. Of these, 147 (34.4%) were livestock owners, and 280 (65.6%) were non-livestock owners. 63 (42.9%) livestock owners, and 104 (37.7%) of non-livestock owners were female. The mean age was 42.6 and 35.9 for livestock owners and non-livestock owners, respectively (Table 4). The crude CCHFV seroprevalence was 4.0% (*n* = 17/427) by IDvet and 2.3% (*n* = 10/427) by Luminex. Among livestock owners, seroprevalence was 6 (4.1%) of 147 by IDvet and 11 (7.5%) of 147 by Luminex. By contrast, among non-livestock owners, seroprevalence was 11 (3.9%) of 280 participants by IDvet and 5 (1.8%) of 280 participants by Luminex (Table 5). Of 147 samples from livestock owners, 139 (94.6%) were double-negative, and 3 (2.0%) were double positive. Among 280 samples from non-livestock owners, 268 (95.7%) were double-negative, and 4 (1.4%) were double-positive (Table 6).

**Table 4:** Social demographics by livestock ownership status n = 427.

Sociodemographic characteristics of the febrile patients in Moshi, Tanzania	Total *N* (%)	Livestock owners *N* = 147 *n* (%)	Non-livestock owners *N* = 280 *n* (%)
Age			
≤5	3 (0.7)	0 (0.0)	3 (1.1)
20 to 34	168 (39.3)	44 (29.9)	124 (44.3)
35 to 59	218 (51.1)	83 (56.5)	135 (48.2)
60 to max	38 (8.9)	20 (13.6)	18 (6.4)
Mean age (SD)	38.2 (13.8)	42.6 (13.9)	35.9 (13.2)
Sex			
*Female	167 (39.5)	63 (42.9)	104 (37.7)
Male	256 (60.5)	84 (57.1)	172 (62.3)

SD is standard deviation. *Frequency of sex distribution among non-livestock owners does not sum to total due to missing participant data.

**Table 5:** IDvet and luminex assay CCHFV seroprevalence among livestock owners and non-livestock owners.

Diagnostic test	Livestock owners (*N* = 147) *n* (%; 95 CI)	Non-livestock owners (*N* = 280) *n* (%: 95 CI)	Total (*N* = 427) *n* (%; 95 CI)
IDvet			
Negative	141 (95.9; 91.4–98.1)	269 (96.1; 93.1–97.8)	410 (96.0; 93.7–97.5)
Positive	6 (4.1; 1.9–8.6)	11 (3.9; 2.2–6.9)	17 (4.0; 2.5–6.3)
Luminex			
Negative	142 (96.6; 92.3–98.5)	275 (98.2; 95.9–99.2)	417 (97.7; 95.7–98.7)
Positive	5 (3.4; 1.5–7.7)	5 (1.8; 0.8–4.1)	10 (2.3; 1.3–4.3)

**Table 6:** CCHFV IDvet and luminex assay test result combinations among febrile patients in northern Tanzania.

Diagnostic test results combination	Livestock owners frequency n/*N* (%)	Non-livestock owners frequency n/*N* (%)
IDvet (+) luminex (+)	3/147 (2)	4/280 (1.4)
IDvet (+) luminex (−)	3/147 (2)	7/280 (2.5)
IDvet (−) luminex (+)	2/147 (1.4)	1/280 (0.4)
IDvet (−) luminex (−)	139/147 (94.6)	268/280 (95.7)

+ is test positive; − is test negative.

### Median posterior estimates for seroprevalence, sensitivity and specificity, of IDvet and Luminex

Both assays showed comparable sensitivities. The median posterior sensitivity of the IDvet assay was 0.893 (95% BCI: 0.555–0.996), while the Luminex median posterior sensitivity estimate was 0.884 (95% BCI: 0.541–0.995). Specificity estimates were high for both assays. The IDvet assay had a median posterior specificity of 0.972 (95% BCI: 0.936–0.996). The Luminex assay exhibited a higher median posterior specificity of 0.987 (95% BCI, 0.947–0.995) (Figure 1). The posterior median seroprevalence of CCHFV was slightly higher among livestock owners (0.027, 95% BCI, 0.002–0.081) than among non-livestock owners (0.017, 95% BCI, 0.001–0.044) (Figure 2). The covariance parameters for exposed and non-exposed individuals were centered near zero, with credibility interval encompassing zero, indicating limited residual dependence between the two assays after adjusting for infection status (Table 7).

**Figure 1:**
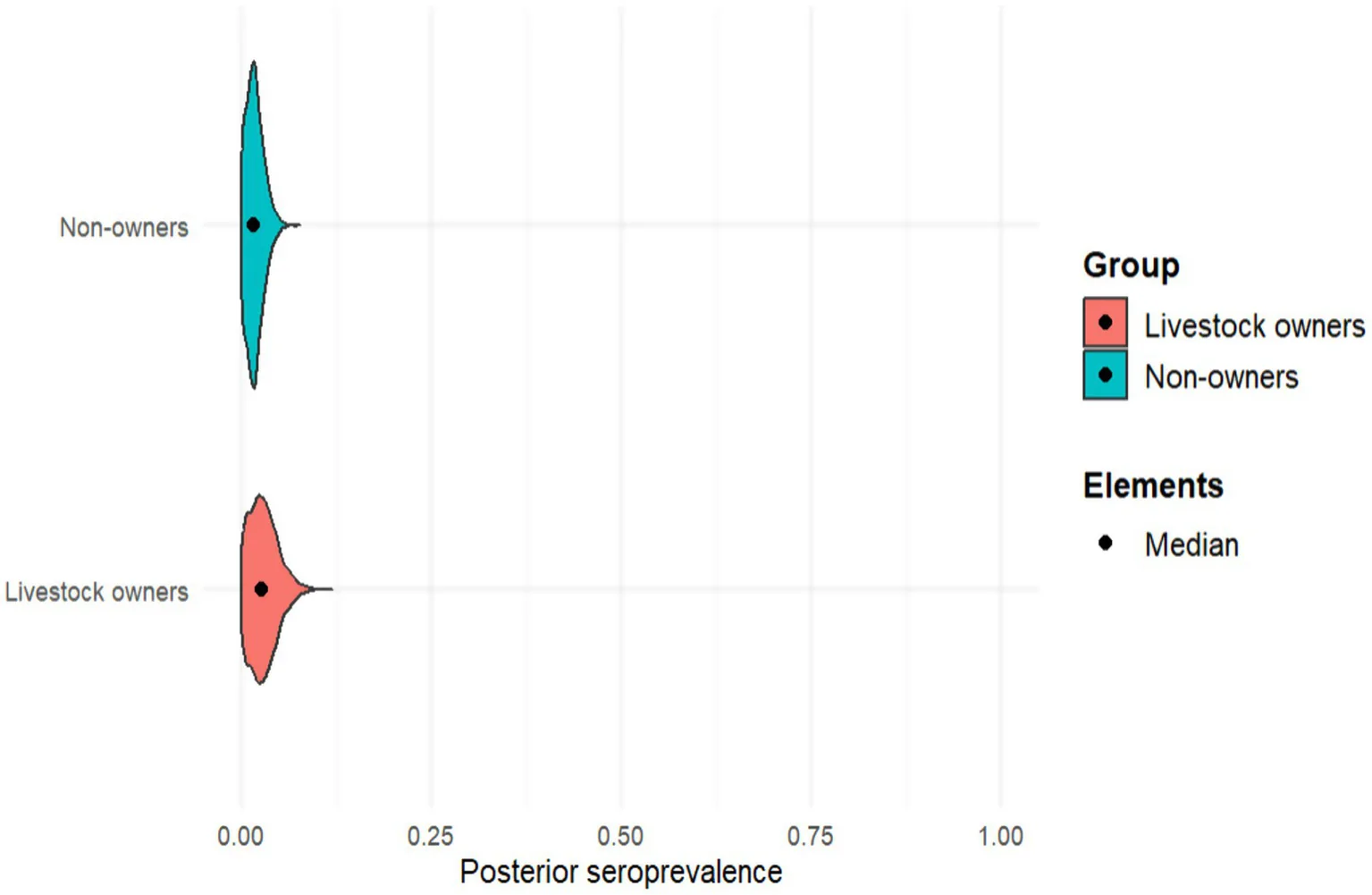
Violin plot of posterior estimates for CCHFV sensitivity and specificity among all participants using IDvet and Luminex assays. Black point represents the median posterior estimate. Colour represents the distribution of posterior samples.

**Figure 2:**
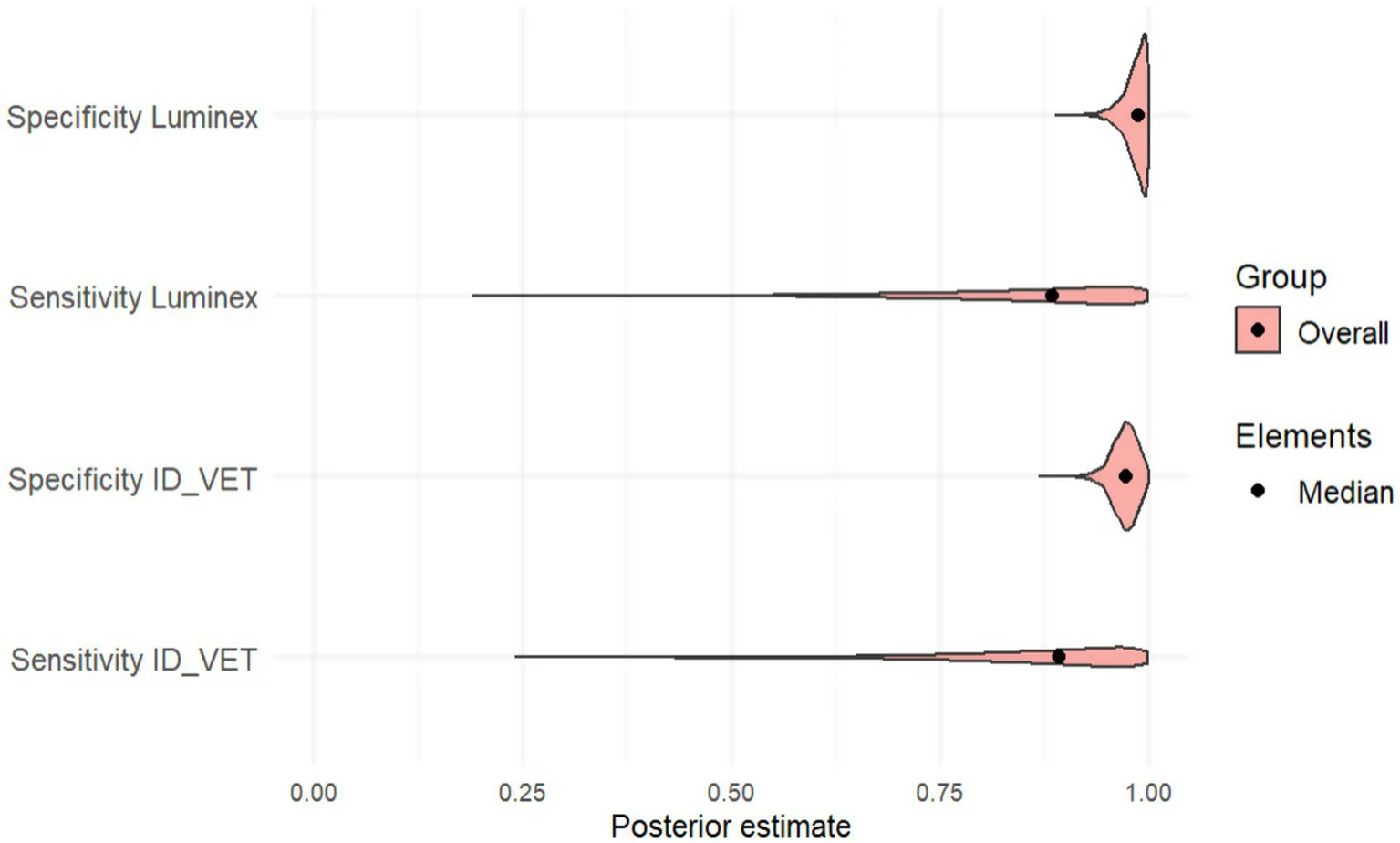
Violin plots of posterior estimates for CCHFV seroprevalence among livestock owners and non-livestock owners. Black dots denotes the posterior median estimate. Colour represents the distribution of posterior samples in livestock owner and non-livestock owner subpopulations.

**Table 7:** Median posterior and 95% Bayesian credibility interval parameter estimates for IDvet and Luminex assays.

Parameter	Median	2.5% BCI	97.5% BCI
Seroprevalence among livestock owners	0.027	0.002	0.069
Seroprevalence among non-livestock owners	0.017	0.001	0.044
Sensitivity IDvet	0.893	0.555	0.996
Specificity IDvet	0.972	0.936	0.996
Sensitivity luminex	0.884	0.541	0.995
Specificity luminex	0.987	0.947	1.000
CovEP	−0.002	−0.124	0.124
CovEN	0.004	−0.009	0.028

CovEN is covariance in exposure negative; CovEP is covariance in exposure positive; BCI is Bayesian credibility interval.

The model had a Gelman-Rubin R-hat ≤1.1 and showed convergence for each parameter. All parameters had an R-hat estimate <1.1 and an effective sample size ≥400 (the minimum threshold), and most exceeded 1,500. The Heidelberger–Welch (H-W) stationarity test passed for all parameters; a few converged after discarding a few early iterations, but they ultimately passed the test, providing evidence of convergence. Trace plots are given in Figure 3. Both assays showed very high NPVs (>0.99) across all groups. The PPVs were lower, particularly for IDvet, while Luminex showed higher PPV among non-livestock owners, consistent with its higher specificity (Table 8).

**Figure 3:**
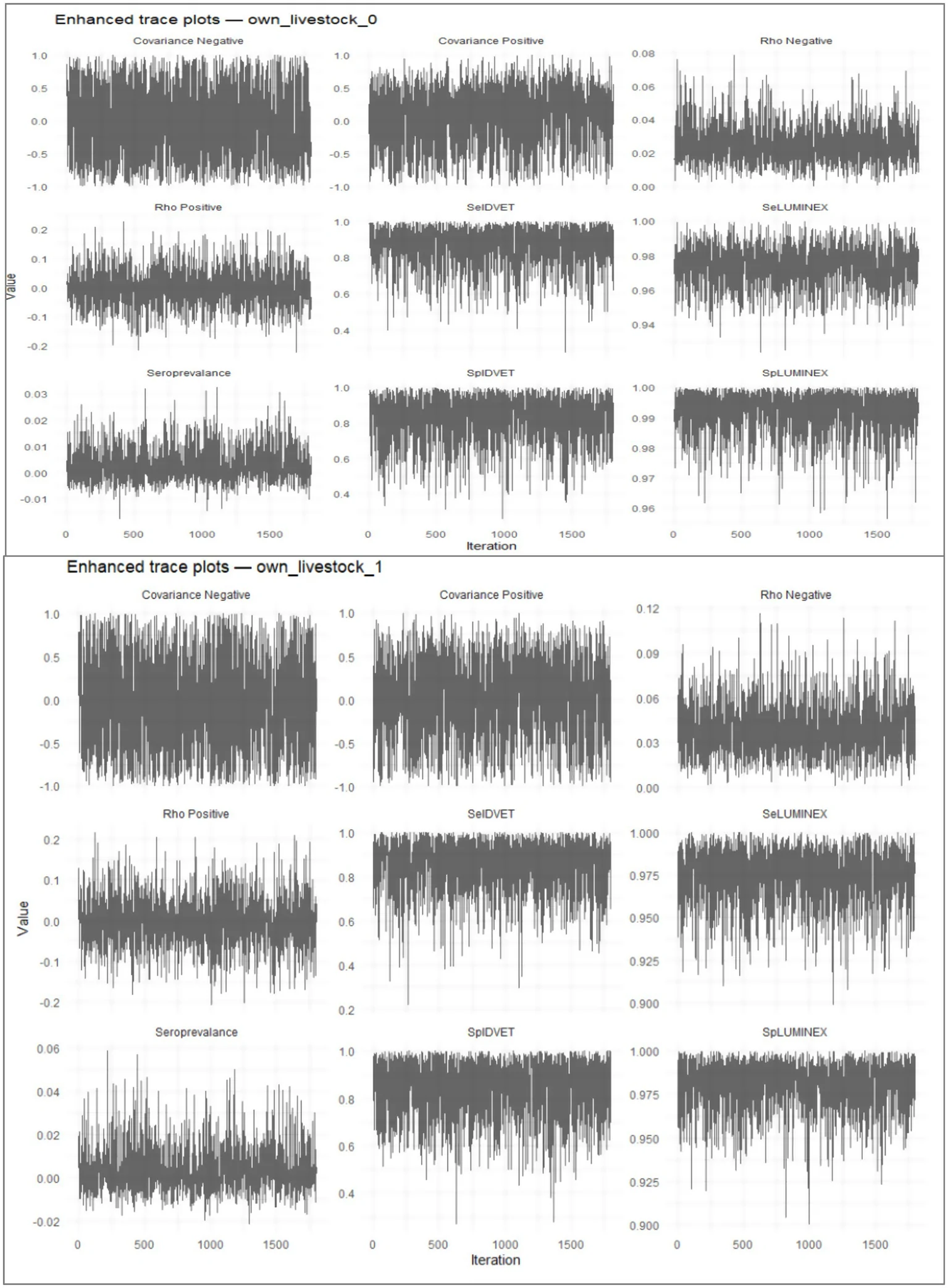
Trace plots of model parameters. Se is sensitivity; Sp is specificity; own_livestock_1 is livestock owners; own_livestock_0 is non-livestock owners.

**Table 8:** Positive and negative predictive values for IDvet and Luminex assays among all participants.

Parameter	Median (95% BCI)
PPV IDvet	0.452 (0.094, 0.910)
NPV IDvet	0.998 (0.987, 0.999)
PPV luminex	0.682 (0.147, 0.986)
NPV luminex	0.997 (0.982, 0.999)

PPV is positive predictive value; NPV is negative predictive value; BCI is Bayesian credibility interval.

## Discussion

We compared the diagnostic performance of the IDvet ELISA and the Luminex microsphere immunoassay for detecting prior CCHFV exposure using a BLCM framework in the absence of a gold standard. The median posterior specificity estimates for IDvet and Luminex assays were high at 0.976 and 0.991, respectively. Sensitivity median posterior estimates for IDvet and Luminex assays were lower and more variable, at 0.897 and 0.869, respectively. Although the posterior estimate for seroprevalence was higher in livestock owners (0.038) than non-livestock owners (0.023), there was overlap in the BCIs for these subpopulations. The slightly higher seroprevalence among livestock owners might be explained by the age difference among livestock owners which is slightly higher, meaning more years of potential cumulative occupational and environmental exposure. The presence of seropositive individuals among non-livestock owners suggests that CCHFV exposure extends beyond groups who have close links with livestock who are typically considered at high risk. One possible explanation is that environmental exposure to infected ticks, handling of contaminated animal products outside of the home, or other unrecognised community pathways may contribute to infection risk. Alternatively, it could be that reports of livestock ownership does not correlate with livestock contact related activities such as milking or assisting deliveries (Cash-Goldwasser et al., 2018).

In the absence of a recognised serological gold standard and limited access to neutralisation assays requiring BSL-4 containment, BLCM provided a robust framework for estimating assay sensitivity and specificity. The findings show that both assays achieved high diagnostic accuracy, with comparable sensitivity and specificity estimates and excellent negative predictive values. These results suggest that both platforms are suitable for CCHFV serosurveillance and epidemiological investigations. Importantly, the study provides evidence supporting the use of the more accessible IDvet ELISA as a practical screening tool in resource-limited settings, while the Luminex platform may offer additional value in specialised reference laboratories and research applications. The principal contribution of this study therefore lies in strengthening the evidence base for CCHFV serological diagnostics in endemic regions where reference-standard testing is not readily available.

Median posterior estimates for sensitivity were moderate for both assays. Although the Luminex assay had a higher median specificity estimate, resulting in a higher PPV than IDvet, our BLCM estimated high specificity (>95%) for both assays. Both assays showed very high NPV, indicating that negative results can confidently rule out previous exposure to CCHFV in both groups. Although sensitivity estimates were similar, the Luminex assay showed slightly higher specificity and PPV than IDvet. Because this study focused on febrile hospital patients rather than community populations, direct epidemiological comparisons should be interpreted cautiously (Atim et al., 2023; Kizito et al., 2024; Mukhaye et al., 2024). In low-resource environments lacking advanced multiplex platforms, IDvet provides a scalable initial screening option. Despite slightly lower specificity, it showed excellent diagnostic performance with high NPVs, making it useful for excluding prior exposure. This supports the strategic use of IDvet as a frontline screening tool in resource-limited settings as compared with Luminex assay which requires more specialised laboratory resources.

A methodological issue was the potential dependence between the two assays, both targeting host antibody responses to the CCHFV NP. Complete independence is biologically unlikely, and ignoring it risks bias. To address this, the study included a covariance parameterisation (Johnson et al., 2001). Covariance estimates were near zero, and credibility intervals crossed zero, indicating limited residual dependence after accounting for infection status. Conventional approaches that compare new assays against imperfect reference tests may underestimate or overestimate diagnostic accuracy(Bodenham et al., 2021). The Hui-Walter framework enabled simultaneous estimation of sensitivity and specificity while accounting for uncertainty in true infection status and conditional dependence between assays(Cheung et al., 2021). This approach is particularly valuable for emerging and neglected zoonotic infections such as CCHFV, where validated reference standards remain limited.

The slightly better overall performance of the IDvet assay might be due to both scientific and operational reasons. IDvet demonstrated marginally higher sensitivity, indicating it detects low-level antibodies more effectively in the low-prevalence febrile group(Balinandi et al., 2021). Its double-antigen ELISA format enhances antibody capture, particularly in individuals with weak or previous exposure to CCHFV. In contrast, the Luminex asssay, which relies on fluorescence and bead interactions, may have lower detection in low-titer sera. Operationally, IDvet offers a simpler workflow with fixed reagents and cutoffs, whereas Luminex involves complex procedures that can introduce variability, especially in resource-limited settings. Although Luminex’s higher specificity reduces false positives, it might decrease sensitivity for weakly reactive samples. Signal competition or background interference can also affect multiplex testing. Overall, IDvet may be better suited for decentralised screening, while Luminex may be more appropriate for confirmatory testing in advanced laboratories(Sas et al., 2018). At the country level the IDvet can be used by the Tanzania Veterinary Laboratory Agency and human health facilities in tandem to provide a unified data stream for national CCHF preparedness and outbreak response.

Several limitations should be acknowledged when interpreting this comparison between assays. First, the relatively small number of seropositive samples as the present analysis required an adequate volume of serum specimens as we used archived serum samples, we had no control over them which lead to wide BCIs particularly for sensitivity and seroprevalence estimates. Therefore this analysis could be improved with a larger sample size, more data would improve the posterior estimate accuracy and reduce BCIs. Second, both assays measured IgG antibodies and therefore reflected prior exposure rather than acute infection. Third, prior distributions were informed by a limited number of published diagnostic studies, many of which differed in study design, population characteristics, and reference methods. Finally, test samples were archived sera collected in 2012–2014, and the current epidemiological status of CCHFV may have differed. Further analyses would benefit from including more recently collected samples.

Overall, the findings indicate that both IDvet and Luminex assays are suitable for CCHFV serosurveillance, each with distinct advantages. The Luminex assay demonstrated superior specificity and may be preferable for confirmatory or research applications, whereas the IDvet assay offers greater practicality for routine screening and surveillance in resource-limited settings. Future studies with larger sample sizes, molecular confirmation, and evaluation across diverse endemic populations will be important for further refining diagnostic performance estimates and supporting harmonisation of CCHFV serological testing strategies.

## Conclusion

This study demonstrates that both the IDvet ELISA and the Luminex microsphere immunoassay are highly accurate in detecting previous CCHFV exposure, as evaluated by a BLCM that does not rely on a serological gold standard. Both tests exhibit high specificity and NPVs, making them dependable tools for ruling out prior exposure in settings with low disease prevalence. These characteristics make them well-suited for routine screening and serosurveillance, especially in resource-constrained environments. The Bayesian latent class approach provided reliable estimates for test performance, accounting for uncertainties in true infection status and potential reliance between assays. This research offers essential evidence to inform CCHFV serological surveillance and diagnostics in endemic regions lacking reference-standard tests. Future studies with larger sample sizes, molecular validation, neutralisation assays where applicable, and varied population assessments will enhance diagnostic accuracy and promote a harmonised One Health approach to CCHFV surveillance.

## Data Availability

The original contributions presented in the study are included in the article/Supplementary material, further inquiries can be directed to the corresponding author.
