# Supplementary material for: Comparison of two Crimean-Congo haemorrhagic fever virus serological tests in Tanzanian febrile patients using a Bayesian latent class approach
Source: Front Microbiol. 2026 Oct 16;17:1894712. doi: 10.3389/fmicb.2026.1894712 (PMC13719626; doi:10.3389/fmicb.2026.1894712)
Supplement: Supplementary file 1 [file Data_Sheet_1.DOCX]

**Results for different priors**

**First set of priors**

**All set at beta(1,1)**

| **Test and parameter** | **Prior Distribution Beta (α, β)** |
| --- | --- |
| ID-VET Sensitivity | Beta (1,1) |
| ID-VET Specificity | Beta (1,1) |
| IFA Sensitivity | Beta (1,1) |
| IFA Specificity | Beta (1,1) |
| Disease Prevalence | Beta (1,1) |

**Median posterior and 95% Bayesian credibility interval parameter estimates for IDvet and Luminex aessays**.

| **Parameter** | **Median** | **Lower_95CI** | **Upper_95CI** |
| --- | --- | --- | --- |
| Seroprevalence among livestock owners | 0.105 | 0.007 | 0.991 |
| Seroprevalence among non-livestock owners | 0.938 | 0.005 | 0.997 |
| Sensitivity IDvet | 0.061 | 0.004 | 0.929 |
| Specificity IDvet | 0.941 | 0.072 | 0.996 |
| Sensitivity Luminex | 0.047 | 0.002 | 0.910 |
| Specificity Luminex | 0.954 | 0.099 | 0.998 |
| CovEP | 0.011 | -0.140 | 0.176 |
| CovEN | 0.013 | -0.137 | 0.169 |

**Second set of priors**

Beta values for sensitivity of Luminex as beta(1,1) living other priors as previous

| **Test and parameter** | **Beta distribution (α, β)** |
| --- | --- |
| IDvet sensitivity | (6.5, 1) |
| IDvet specificity | (2, 1) |
| Luminex sensitivity | (1, 1) |
| Luminex specificity | (2, 1) |
| Disease seroprevalence | (2, 11) |

**Median posterior and 95% Bayesian credibility interval parameter estimates for IDvet and Luminex aessays**.

| **Parameter** | **Median** | **Lower_95CI** | **Upper_95CI** |
| --- | --- | --- | --- |
| Seroprevalence among livestock owners | 0.041 | 0.010 | 0.092 |
| Seroprevalence among non-livestock owners | 0.032 | 0.008 | 0.072 |
| Sensitivity IDvet | 0.891 | 0.543 | 0.995 |
| Specificity IDvet | 0.982 | 0.945 | 0.999 |
| Sensitivity Luminex | 0.475 | 0.042 | 0.965 |
| Specificity Luminex | 0.986 | 0.940 | 0.999 |
| CovEP | 0.008 | -0.138 | 0.163 |
| CovEN | 0.004 | -0.008 | 0.031 |

**Third set of priors**
In this set the Beta values for sensitivity of Luminex was set at beta(1,1), changes is made on the prior for IDvet sensitivity and seroprevalence the parameters using less concentrated/non-informative priors

| **Test and parameter** | **Beta distribution (α, β)** |
| --- | --- |
| IDvet sensitivity | (5, 1) |
| IDvet specificity | (2, 1) |
| Luminex sensitivity | (1, 1) |
| Luminex specificity | (2, 1) |
| Disease seroprevalence | (1, 10) |

**Median posterior and 95% Bayesian credibility interval parameter estimates for IDvet and Luminex aessays**.

| **Parameter** | **Median** | **Lower_95CI** | **Upper_95CI** |
| --- | --- | --- | --- |
| Seroprevalence among livestock owners | 0.031 | 0.002 | 0.083 |
| Seroprevalence among non-livestock owners | 0.023 | 0.002 | 0.063 |
| Sensitivity IDvet | 0.869 | 0.477 | 0.995 |
| Specificity IDvet | 0.977 | 0.937 | 0.998 |
| Sensitivity Luminex | 0.491 | 0.035 | 0.968 |
| Specificity Luminex | 0.984 | 0.938 | 0.999 |
| CovEP | 0.006 | -0.158 | 0.174 |
| CovEN | 0.007 | -0.006 | 0.034 |

**Insights from the analysis**

Both models using the second and third sets of priors achieved convergence, and the posterior estimates were very similar. However, the third set of priors represents less concentrated (less informative) prior distributions compared with those used in the second model.

When the prior for Luminex sensitivity was changed to Beta(1,1), the posterior estimate changed to 0.491 with a 95% Bayesian credible interval (BCI) of 0.035–0.968. This credible interval is considerably wider than the previous estimate, indicating greater uncertainty around the Luminex sensitivity parameter when a non-informative prior is used. This suggests that the available data provide limited information for estimating Luminex sensitivity.

**Explanation of Prior selection**

Prior distributions for all model parameters were specified as weakly informative Beta(α, β) distributions. Rather than deriving priors directly from the point estimates and confidence intervals reported in the literature using the epi.betabuster function, weakly informative priors were adopted that reflects the general direction of the available evidence, that is high sensitivity, high specificity, and low-to-moderate seroprevalence while allowing sufficient uncertainty for the observed data to meaningfully influence the posterior estimates. This approach was chosen because several literature estimates were derived from single studies with moderate sample sizes, and some estimates were reported at the boundary value of 100%, which yielded highly concentrated priors implying near-certainty when parameterized using epi.betabuster.

For the IDvet assay, Sas et al. (2018) reported a sensitivity of 98.9% (95% CI: 96.8–99.8%) based on 257 samples. Using this estimate and its confidence interval directly to derive a Beta prior resulted in a highly informative distribution with large α and β parameters. Therefore, a weaker prior, Beta(6.5,1), was specified to reflect the high sensitivity reported in the literature while allowing the observed data to contribute substantially to posterior estimation. Similarly, the reported specificity of 100% (95% CI: 99.8–100%) represents a boundary value that produces excessively concentrated Beta priors. Consequently, a weakly informative Beta(2,1) prior was adopted to preserve the assumption of high specificity while allowing uncertainty. No published estimates of Luminex sensitivity for human CCHFV serology were identified. Therefore, a non-informative Beta(1,1) prior was specified for this parameter. The reported specificity was 100% (95% CI: 98.0–100%) based on 182 samples (Wu et al., 2014). Following the same rationale used for the IDvet specificity prior, a weakly informative Beta(2,1) distribution was specified.

Seven studies conducted in East Africa reported CCHFV seroprevalence estimates ranging from 1.8% to 19% (table2). Rather than specifying a mode-matched prior, we used a weakly informative Beta (1,10) distribution with a prior mean of 9.1%, which approximates the sample-size-weighted pooled seroprevalence estimate of 7.0% from these studies. This distribution assigns greater probability to lower prevalence values while retaining reasonable support for the higher values reported in the literature. Consequently, the prior is sufficiently weak to allow the observed data, rather than previous studies, to primarily influence the posterior estimates
